# Unraveling the biophysical underpinnings to the success of multispecies biofilms in porous environments

**DOI:** 10.1038/s41396-019-0381-4

**Published:** 2019-03-04

**Authors:** David Scheidweiler, Hannes Peter, Paraskevi Pramateftaki, Pietro de Anna, Tom J. Battin

**Affiliations:** 1 Stream Biofilm and Ecosystem Research Laboratory, Ecole Polytechnique Fédérale de Lausanne, CH-1015 Lausanne, Switzerland; 2 Institute of Earth Sciences, University of Lausanne, CH-1015 Lausanne, Switzerland

**Keywords:** Microbial ecology, Sequencing, Microbial ecology, Sequencing

## Abstract

Biofilms regulate critical processes in porous ecosystems. However, the biophysical underpinnings of the ecological success of these biofilms are poorly understood. Combining experiments with fluidic devices, sequencing and modeling, we reveal that architectural plasticity enhances space exploitation by multispecies biofilms in porous environments. Biofilms consistently differentiated into an annular base biofilm coating the grains and into streamers protruding from the grains into the pore space. Although different flow-related processes governed the differentiation of these architectures, both BB and streamers were composed of similar bacterial assemblages. This is evidence for architectural plasticity. Architectural plasticity allowed for complementary use of the space provided by the grain–pore complexes, which increased biofilm carrying capacity at the larger scale of the porous system. This increase comes potentially at the cost of a tradeoff. Contrasting time scales of oxygen replenishment and consumption, we show that streamers locally inhibit the growth of the BB downstream from the grains. Our study provides first insights into the biophysical underpinnings to the success of multispecies biofilms in porous environments.

## Introduction

Today, it is a common understanding that biofilms are a very successful form of microbial life in most ecosystems [1]. Understanding the ecology of microorganisms in general [2] and of biofilms [3, 4], in particular, entails among other a proper evaluation of their interactions with the physical environment. In porous systems, such as in stream sediments, aquifers, and soils, where biofilms regulate critical functions, the properties of the physical environment typically range over several orders of magnitude [5]. However, the traditional focus of research has been on its macroscopic properties, including porosity, permeability, or pore connectivity [6]. These approaches are effective in providing an overall description of the porous environment, however, they do not capture the spatial heterogeneity of the environment with which the biofilms interact.

A first generation of experiments using bioreactors has highlighted the role of microbial growth for the hydrodynamics in porous environments [7–10]. More recently, the advent of fluidic devices has enabled microbiologists to study, at the microscale, the mechanics of bacterial biofilms in porous [11–14], meandering and branching [15–18] systems. These studies have advanced our knowledge on the effects of laminar flow on biofilm architecture (that is, physical structure), otherwise typically studied in turbulent, open-channel flow in streams [3] and laboratory-scale flow chambers [19]. For instance, experimenting with monospecies biofilms, it was shown that secondary flows around corners induce streamer formation [11, 15, 16] that was originally thought to be limited to turbulent flow [20]. Streamers formed by *Pseudomonas* *aeruginosa* were also shown to locally induce clogging, thereby affecting fluid flow through channel systems [17]. Local clogging by rapidly growing bacterial strains can also redistribute resources delivered with the fluid flow, thereby instigating competition between genotypes [18]. Recent work using pore networks mimicking the space between soil aggregates has revealed the spatial organization of *Pseudomonas* *putida* and *Pseudomonas* *veronii* in niches establishing along resource gradients [13].

Current understanding of biofilm architectural differentiation in porous environments largely rests on mono- or dual-species biofilms [11, 12, 15, 17, 18, 21]. This contrasts the massive diversity with hundreds to thousands of bacterial taxa forming biofilms in nature, and notably in streams [22]. A “co-evolutionary” relationship was postulated between these diverse biofilms and their streambed environment, as biofilms evolve in response to the physical and chemical structure of the streambed, and simultaneously modify this environment by changing its topography, hydrodynamics, and chemical gradients [4]. Diverse biofilms may engage in such a “co-evolutionary” relationship to increase their carrying capacity through niche construction, which is understood as the environmental modifications leading to a growth advantage for the modifying organism as well as for other taxa [23]. Niche construction through architectural differentiation can be considered as an emergent biofilm property [24], in the sense that novel niches arise during the spatial organization of multispecies biofilms. How niche construction and tradeoffs between different architectures possibly affects the carrying capacity of biofilms in porous environments where resources (e.g., space, nutrients) for microbial growth are often limited remains elusive at present. Addressing this question is fundamental. Carrying capacity is a key quantity in ecology because it encapsulates environmental constraints with fitness and biogeochemical fluxes. Furthermore, the ability to compete for access to space, notably to appropriate surfaces, and the subsequent colonization thereof appears to be a major component of the success of the biofilm mode of life [25, 26]. Multispecies biofilms may differentiate through compositional segregation, such as the stratified communities in dental plaque [27] or in benthic stream biofilms [39]. However, multispecies biofilms may also exploit space through architectural plasticity, which reflects the capacity of a community to adopt different structures as a response to environmental cues [28].

We developed a fluidic device to study the local hydrodynamics and related mass transfer as potential physical modulators of multispecies biofilm growth across scales, ranging from individual grain–pore complexes to the porous landscape contained within the fluidic device. Such experimental devices are widely used in microbial ecology because they provide time- and space-resolved insights into processes that would be impossible to capture in natural settings [29–31]. Our fluidic device was designed to simulate processes occurring in low Reynolds flow environments, where viscosity dominates over non-linear inertial forces. Under these conditions, the local fluid velocity has a linear dependence on viscous forces: this implies that the local velocity field scales linearly with respect to the imposed physical conditions, (flow rate or pressure drop), making our device applicable to various situations of low Reynolds flow. To simulate the hydrodynamics in porous systems, fluidic devices can be operated via constant flow [11] or constant pressure [17]. We applyed a constant flow as it occurs during base flow when the benthic interface in streams is exposed to steady water flow over time scales relevant for biofilm growth [4, 32]. We combined experiments, time-lapse microscopy, sequencing, and mathematical models to show that biofilms containing high bacterial diversity predictably differentiate into architectures that enable them to increase space exploitation and biomass. Our study highlights how the architectural plasticity of biofilms, at the scale of grain–pore complexes, benefits biofilm growth at the larger scale of the porous environment, and that this occurs at the cost of a tradeoff between architectures.

## Materials and methods

### Experimental design

**Fig. 1:**
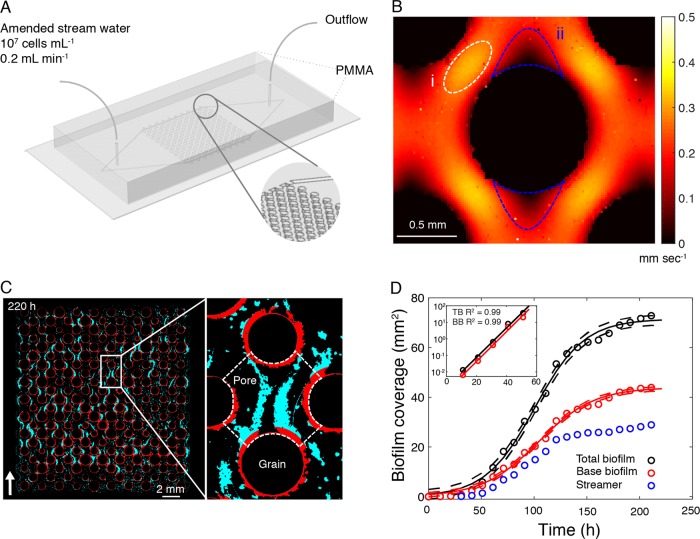
Fluidic device, hydrodynamics, and growth dynamics of biofilms in a porous environment. **a** Design of the fluidic device used to mimic a porous environment for biofilm growth. **b** Characteristic flow fields, as measured by particle image velocimetry in the mid-depth of the fluidics cell; fluid velocity was elevated in the pore throats (i) and reduced on either poles of the grain (ii). **c** Biofilm differentiation into streamers (cyan) and base biofilm (BB; red) across the fluidic device at 220 h. White arrow shows flow direction. **d** Development of biomass as coverage of total biofilm (TB, black circles), and base biofilm (BB, red circles) and of streamers (blue circles) integrated over the porous environment in the fluidic device. TB and BB growth followed a logistic growth (Residual Standard Error = 2.03 and 1.25 for TB and BB, respectively); shown is the logistic model and 95% confidence intervals (solid and dashed lines respectively). Inset: semi-logarithmic plot of biofilm coverage, colored lines represent linear regression

We developed a transparent planar fluidic device (length: 20 mm; width: 20 mm; height: 0.4 mm) mimicking the heterogeneity of grain and pore size encountered in sandy streambeds (Fig. 1a; Fig. S1). The device contains a matrix of 200 pillars (that is, the grains) of various diameter (0.4–1.4 mm) following a Gaussian distribution (Fig. 1a; Fig. S1). The porous landscape was produced with high-precision micromilling (WF31SA, Mikron) in a polymethyl methacrylate (PMMA) layer and sealed with a top PMMA layer and a nitrile O-ring to make the system gas-tight.

The inoculum consisted of streamwater (Avançon, Switzerland) that we filtered (1 µm, Whatman) to remove major grazers. We amended the filtered streamwater with Luria-Bertani broth (diluted 1:3000, yielding a concentration of dissolved organic carbon of 3.4 mg C L^−1^), which contains a diverse mix of organic and inorganic compounds that can be metabolized by multispecies assemblages. The inoculum was contained in initially sterile Polypropylene copolymer bottles (Nalgene) equipped with a sterile air-vent filter, and perfused through the fluidic devices (Minipuls-3 Pump, Gilson Inc.) at a flow rate of *Q* = 0.2 ml min^−1^, corresponding to an average fluid velocity of 0.35 mm s^−1^ (as measured with PIV, see Supplementary material) typical of soil like systems. All components were connected via Tygon tubing (1.02 mm ID; Ismatec) with traps that prevented gas bubbles to enter the fluidic device. Experiments were performed in darkness to avoid phototrophic growth and at room temperature (20 °C). Bacterial abundance in the inoculum was determined (0, 24, 48, 72, and 200 h) using flow cytometry (NovoCyte, ACEA Biosciences) on cells stained with Syto13 (Molecular Probes, Invitrogen).

### Image processing

Time-lapse imaging was performed with an automated transmitted light Zeiss AxioZoom v16 microscope (Plan Neo Fluar Z 1 ×/0.25, FWD 56 mm objective) equipped with a CCD camera (Axiocam 506 mono, Zeiss) and controlled by the Zen 2011 software. Recording every hour a large image composed of 120 pictures (as 12 × 10 tiles), we scanned the area of the entire fluidic device (4 × magnification, 1.01 µm pixel^−1^ resolution). Individual pictures were taken, in the middle horizontal plane of the device, using bright-field microscopy at an exposure time of 100 milliseconds. We extracted architectural information from biofilm areal coverage as a proxy of biomass, as commonly accepted in biofilm studies with microfluidics [17, 18, 33, 34].

In brief, we subtracted the first image (*T* = 0 h) from each subsequent image as background correction. After binarization, we identified a base biofilm (BB) as the fraction of biofilm present in a ring around a grain such that at least 35% of the area is covered by biomass (Supplementary material, Fig. S10A and B*)*. BB thickness was determined with 0.1° increments around each grain. BB perimeter, *P*_*BB*_, was computed as the sum of the Euclidean distances between neighboring points of its external boundary. BB tortuosity was measured as the ratio between the measured *P*_*BB*_ and the grain perimeter (Fig. S10F). Streamers initially occurred as individual small clusters connected by a non-visible expanded polystyrene (EPS) filament. To delineate streamers, clusters distant less than 45 µm were connected into a single streamer (Fig. S11). After skeletonization, streamer length (*L*) was measured as the longest connected path, whereas streamer width (*W*) was derived from Euclidean distance transformation (Supplementary material). Streamer porosity was measured as the percentage of biomass within each streamer boundary (Fig. S11A). For a more exhaustive explanation see Image processing in Supplementary material.

### Flow field characterization

Fluid velocity was determined in the fluidic device devoid of biofilm using microscale particle image velocimetry with fluorescent particles (1 µm; Thermofisher Fluoromax B0100) [35]. A set of four consecutive images, taken every 50 ms, was captured at each location using an exposure time of 5 ms, much smaller than the characteristic time of displacement of the fastest particles over few pixels. Local fluid velocities were derived from measurements of autocorrelation of particle displacement accounting for the time interval between pair of images.

### Biofilm community composition

At the end of the experiment, we sampled the inoculum, BB, and streamer biomass for sequencing (Illumina Miseq platform). In brief, we sampled BB and streamers under the dissection microscope using sterile tweezers and needles. DNA was extracted using the DNeasy Powersoil Kit (Qiagen), and DNA quality, integrity and yield were assessed. DNA was amplified using the 515 F/806 R primers [36] and technical triplicates of each sample were mixed; amplification was verified on an agarose gel and products were further processed for library preparation and sequencing (University of Lausanne). Paired-end sequences were merged, quality-filtered, aligned, and clustered into operational taxonomic units (OTUs) based on 97% sequence similarity using the USEARCH v10 algorithm [37]. Chimeric sequences were removed and taxonomy was assigned to the remaining OTUs using the RDP v16 reference. Singletons were removed and the dataset was rarefied to 5000 sequences. A maximum likelihood phylogenetic tree using nearest-neighbor interchange and the Tamura-Nei substitution model was calculated using Mega6 and displayed using the interactive Tree of Life. We calculated pairwise similarities between the BB and streamer assemblages from OTU relative abundances using Bray–Curtis (abundance weighted) and Jaccard similarities (presence-absence). Bootstrapped confidence intervals were calculated from 1000 randomizations of the incidence matrix.

## Results

### Architectural differentiation of biofilms in porous systems

At the onset of the experiment, laminar flow around the grains generated heterogeneous flow fields across the landscape within the fluidic device, as investigated by particle image velocimetry. Fluid velocity was highest in the center of pore throats and minimal on either pole of the grains along the direction of the fluid flow (Fig. 1b). The porous system was continuously exposed to streamwater containing 375 bacterial OTU at an average cell density of 10^7^ cells mL^−1^ (see Methods). Members of *Comamonadaceae*, *Pseudomonadaceae*, *Caulobacteraceae*, *Oxalobacteraceae,* and *Flavobacteriaceae* were among the most abundant OTUs in the inoculum.

We quantified biofilm growth and architectural differentiation over 220 h using time-lapse microscopy and biofilm coverage as a proxy for total microbial biomass (see Image processing in Supplementary material). Biofilm growth was initiated by bacterial cells forming an annular BB around the grains (Fig. 1c). After 30 h, streamers started to develop downstream from the grains where fluid velocity was elevated (Fig. 1b). Streamer formation under laminar flow has been associated to secondary flows, that arise as soon as the streamlines deviate from a rectilinear to a curvilinear path [15]. Using numerical simulations (COMSOL Multiphysics), we were able to confirm the presence of such secondary flows around the grains in our fluidic device (Fig. S3).

The architectural differentiation of biofilms into BB and streamers was highly reproducible and this independent of the starting inoculum and spatial configuration of the grains within the fluidic devices (Fig. S2). Although we focused on stream biofilms here, we reproduced the experiment with lake water and even with *Pseudomonas putida*, and consistently found the same patterns. During our experiment with streamwater bacteria, all grains (*n* = 200) became coated with BB and 87% of the pores (*n* = 191) contained streamers. On average, BB contributed more to the total biofilm coverage (67 ± 6%) than streamers (34 ± 6%) across all three replicates. At the end of the experiment (220 h), BB covered on average 15 ± 7% and streamers covered on average 9 ± 7% of the space across all pores within the fluidic device.

The temporal dynamics of total biofilm biomass, as the sum of BB and streamers, followed a logistic growth model (Fig. 1d). We assumed biological (e.g., cell reproduction and EPS production) rather than physical (e.g., filtration) processes to drive the logistic growth of BB (Fig. 1d). This is intuitive because the laminar flow (Fig. S4A) around the grains would discourage the filtration by interception of bacterial cells from the bulk fluid. The logistic growth model does not take into consideration cell detachment from BB, which is likely negligible in a rather steady laminar flow (Fig. S5). After an inflection point at 110 h, BB reached higher carrying capacity than the streamers and contributed the majority of the biofilm biomass contained within the fluidic device. In contrast, streamers are known to filter particles [3] and bacteria [17] by interception from the bulk fluid, a process that enhances streamer increase in size. Using neutrally buoyant fluorescent microspheres, we were able to confirm such filtration by streamers in our fluidic devices (see Filtration model in Supplementary material; Fig. S4). We therefore assumed that filtration dominated streamer growth and did not fit a logistic model to their growth curve (Fig. 1d). The growth of streamers reached a plateau after 120 h, coinciding with the point in time when their porosity reached a minimum (see below).

### Community composition

**Fig. 2:**
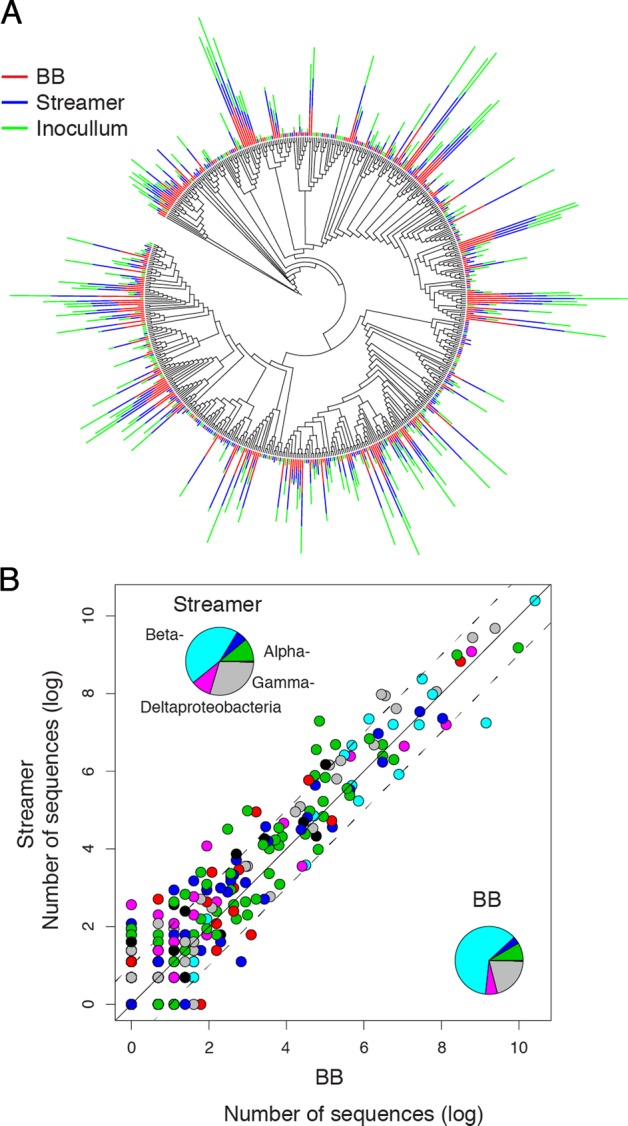
Similar community composition in BB and streamers. **a** Phylogenetic tree presenting the 16 S rRNA gene diversity sampled from streamers, BB and the inoculum. The bars show the relative abundance (log-transformed) of OTUs in streamers (blue), BB (red), and the inoculum (green). Abundant OTUs are found among diverse taxonomic groups and these abundant OTUs are generally found in similar abundances in each community. **b** Abundant OTUs in the base biofilm tended also to be abundant in the streamer. Each point indicates an OTU, the color reflect its respective taxonomic affiliation as shown in the inserts. OTUs tend to fall along the 1:1 line (solid line, the dashed lines border the regions in which the abundance of an OTU is two times larger in the respective architecture). Both biofilm architectures were dominated by OTUs classified as Beta- and Gammaproteobacteria

Strikingly, the architectural differentiation into BB and streamers was not reflected by pronounced differences in community composition as revealed by sequencing of the 16 S rRNA gene. BB and streamers had 206 OTUs in common accounting for 99.9% of the 275,912 sequences in these samples, whereas 85 and 136 OTUs were unique to BB and streamers, respectively. All abundant OTUs (>12 sequences) were shared between both architectures, whereas non-shared OTUs were generally rare, represented by only a few sequences (Fig. S6A). Taxonomic diversity was relatively high (Fig. 2a), including 32 bacterial phyla and four archaeal phyla. Most OTUs belonged to the beta-, gamma-, and alpha-proteobacterial classes, accounting for 90.6% of the sequences in BB and for 84.4% of the sequences in streamers. Beta-Proteobacteria represented 61.5% of the sequences in BB and 44.6% of the sequences in streamers. GammaProteobacteria accounted for 20.6 and 29.2% of the sequences in BB and streamers, and alpha-Proteobacteria accounted for 8.5% and 11.1% of the sequences in BB and streamers, respectively. Both, at family and at OTU level there were no marked differences in community composition between BB and streamers (Fig. 2b*)*. Relative abundance in streamers was highly correlated with relative abundance in BB on family (*r* = 0.94, *n* = 98) and OTU level (*r* = 0.92, *n* = 427).

Moreover, both biofilm communities were similarly recruited from the inoculum with 96.1% of the abundant OTUs in BB and 98.3% of abundant OTUs in streamers being found in the inoculum (Fig. S6A). Most of these OTUs were detected in similar relative abundance in the biofilm and inoculum; however, those that were enriched in biofilms compared to the inoculum were enriched to similar degrees in BB and streamers (Fig. S6B). Based on presence/absence of OTUs in a rarefied dataset, Jaccard similarity (*J*) between BB and streamers was high (0.89, bootstrapped 2.5–97.5 confidence intervals, CI: 0.85–0.94). Taking into account the abundance of OTUs, Bray–Curtis (BC) similarity between streamers and BB was somewhat lower (0.75, CI: 0.62–0.87). The streamer community was more similar to the community in the inoculum (*J* = 0.90, CI: 0.86–0.94; BC = 0.74, CI: 0.59–0.83) than was the BB community (*J* = 0.83, CI: 0.78–0.89; BC = 0.58, CI: 0.38–0.74).

### Controls on BB growth

**Fig. 3:**
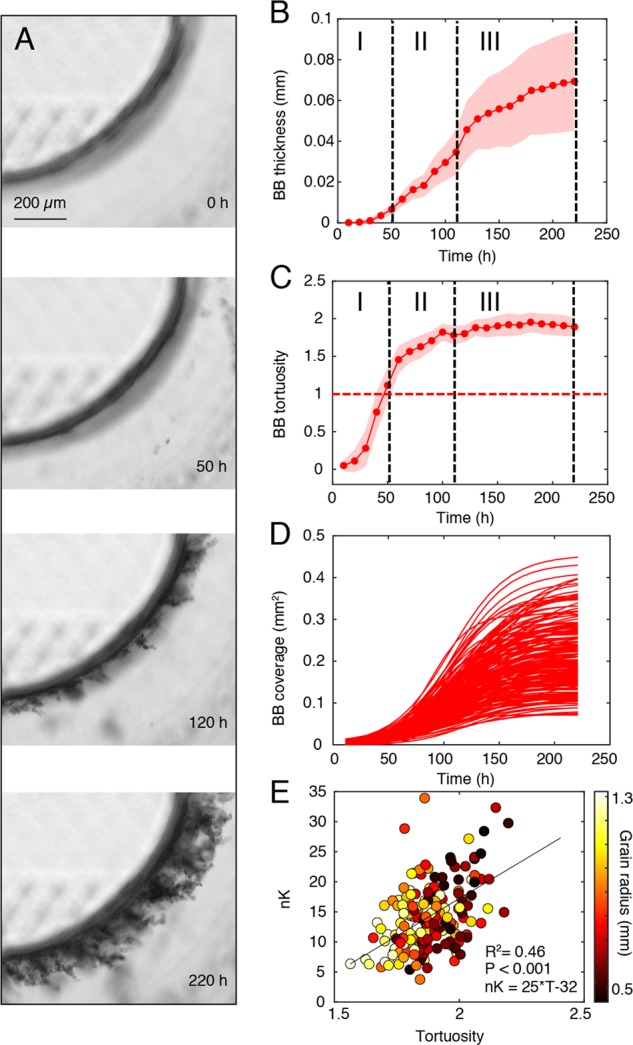
Growth dynamics of base biofilm (BB) features. **a** Micrographs showing the development of BB from a thin layer (50 h) into finger-like structures (beyond 120 h) translating into increased tortuosity. **b**, **c** Dynamics of BB thickness and tortuosity; shown is the average and standard deviation (SD; shaded area) from BB coating 200 grains. BB growth in thickness exhibited three distinct phases, highlighted by black dashed lines (I–III); BB-coated grains during phase I, further grew in thickness during phase II to asymptotically reach a plateau during phase III. BB tortuosity equaled 1 (red dashed line) at 50 h when BB formed a closed layer around the grains and further increased during phase II to level off in phase III. Changes in the growth dynamics of BB thickness corresponded to changes in tortuosity (red dashed line shows tortuosity = 1). **d** Modeled BB coverage for individual grains (*n* = 200) showed a logistic growth, which was used to infer carrying. **e** Normalized carrying capacity (nK) versus BB tortuosity at 220 h (for all the 200 grains); color-coding denotes grain diameter (mm)

We identified three distinct growth phases for BB based on coverage, thickness and tortuosity, all measured on the xy plane. Tortuosity was determined as the ratio between the actual BB perimeter and the grain perimeter (Fig. 3a and S10; see Image processing in Supplementary material). During the first 50 h, BB thickness increased exponentially as expected for cellular growth (doubling time 6.5 h) released from resource constraints. Tortuosity increased from values < 1 before the grain surface was entirely colonized, to 1 when the grain surface was blanketed by a thin biofilm (Fig. 3a–c). A second phase was characterized by growth in thickness concomitant with its differentiation into finger-like structures, which further increased tortuosity (>1). After 120 h, both thickness and tortuosity tended asymptotically towards a plateau (Fig. 3a–c, Fig. S7). Tortuosity enlarges the biofilm surface area and hence mass transfer of nutrients and resources to the biofilm; it was therefore suggested that tortuosity facilitates biofilm growth even in nutrient-depleted environments [3]. We hypothesized that tortuosity increased BB carrying capacity in the porous environment within our fluidic device. This was indeed supported by the positive relationship (*R*^2^ = 0.48, *p* < 0.001) between the dimensionless, normalized carrying capacity, n*K*, (see Carrying capacity in Supplementary material) and tortuosity (Fig. 3e). In addition, n*K* tended to be higher for BB coating smaller grains (*R*^2^ = 0.28, *p* < 0.001), which displayed higher tortuosity (*R*^2^ = 0.43, *p* < 0.001) (Fig. 3e).

### Controls on streamer growth

**Fig. 4:**
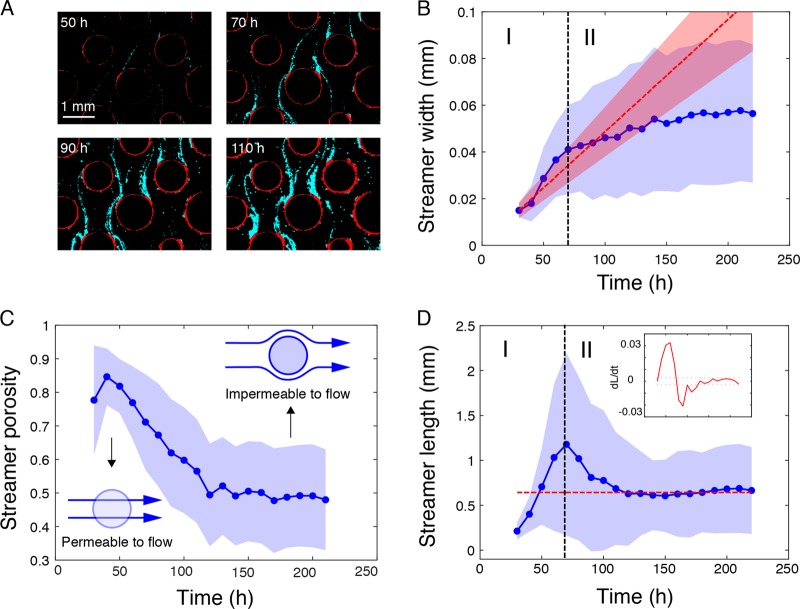
Dynamics of streamer features. **a** Color-coded images showing streamer (cyan) development at the scale of individual grain–pore complexes. **b**–**d** Dynamics of streamer architectural parameters; shown is the average and standard deviation (blue dots and shaded area respectively) from the initial 7 to the final 328 streamers. **b** The initial phase of streamer width development was well captured by a filtration model (red dashed line, while shaded area shows uncertainty interval) parameterized by empirical measurement of filtration the efficiency β. **c** Streamer porosity decreased with time, which assumedly deviated fluid flow and transported bacterial cells, thereby reducing filtration. **d** Dynamics of streamer length initially increasing linearly until 70 h to decrease and reach a quasi-constant length scale after 120 h, highlighted by the red dashed line (inset shows the dynamics of the streamer length changes)

To infer biophysical controls on streamer growth, we deconvoluted the growth of each streamer (*n* increasing from 7 to 328 streamers during biofilm growth) into its components of average width (*W*) and total length (*L*) (see Image processing in Supplementary material). Streamers emanated from initially thin filaments—visible from 30 h on and putatively consisting of EPS—that extended into the free pore space (Fig. 4a). After 220 h of growth, we regularly found two streamers per grain protruding into the downstream pore space. Based on the assumption that filtration of bacterial cells from the bulk liquid contributes to streamer growth in width, we present here the following model to estimate streamer width, *W*. We infer *W* from (2*βvCVt*)/π, where *β* is the filtration efficiency (fraction of bacterial cells removed by the streamer), *v* is the average pore fluid velocity, *C* is cell concentration in the bulk liquid, *V* is the individual bacterial cell volume, and *t* is time (see Filtration model in Supplementary material). Values of *W* modeled with an empirical *β* (0.026 ± 0.027) were well bracketed by measured values during an initial linear increase of *W* (Fig. 4b). After this linear increase, observed values of *W* deviated from model predictions and reached a plateau (0.04 ± 0.02 mm) ~ 70 h (Fig. 4b). We attribute this deviation to a decrease in filtration capacity of the streamers as porosity and hence permeability to the fluid flow is reduced (Fig. 4c). This notion is supported by the observation that streamers with reduced porosity deviated flow and transported cells (or particles) around the streamers themselves (Fig. S4B).

Streamer growth in length was characterized by a conspicuous over-shooting at 70 h (Fig. 4d). After an initial linear elongation to *L* = 1.2 ± 1.0 mm, streamer *L* abruptly decreased and reached a quasi-equilibrium at *L* = 0.59 ± 0.46 mm (Fig. 4d, inset). The change point at 70 h coincided with an average width reduction of the pore throat by 5.5 ± 2% (evaluated over 360 pore throats) as induced by BB growth.

### Mass transfer limitation causes inhomogeneous distribution of BB

**Fig. 5:**
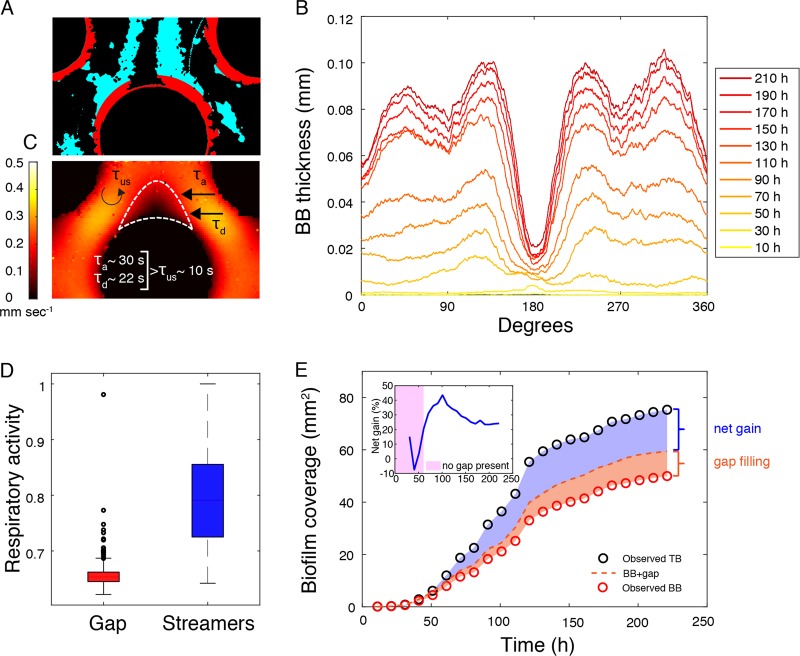
Biofilm architectural differentiation is associated with tradeoffs in biomass allocation. **a** Color-coded picture showing BB with a gap between both streamers at the downstream pole of a grain. **b** The dynamics of the angular distribution of BB thickness supports the consistency of a biomass gap downstream of the grains, which emerged in concurrence with streamer maximum width ~ 70 h (see Fig. 4). **c** Time scales of oxygen diffusion (*Τ*_d_), advection (*Τ*_a_), and in-streamer oxygen uptake (*Τ*_us_) suggest that the gap results from oxygen limitation. **d** Biofilm respiratory activity (determined as the relative formazan fluorescence per unit of biofilm surface, Methods) was higher in the streamers than in the reduced BB within the gap. Box plots show mean and error bars indicate data range within 5th and 95th percentiles. **e** Comparison of total biofilm (TB) coverage with base biofilm (BB) coverage with and without gap, showing that streamers increase the carrying capacity of biofilms in the porous system. The inset shows the net gain (%) owing to streamer differentiation from the appearance of streamers on (when no gap was present in BB) and defined as (streamer – gap filling)/(BB + gap filling) 100

The annular growth of BB did not completely coat the grains (Fig. 5a). Rather, the angular distribution of BB biomass revealed a gap that consistently emerged downstream of the grains and between the streamers from 70 h on (Fig. 5b).

We hypothesize that this gap results from resource limitation of BB owing to low mixing and elevated bacterial activity within the streamers reducing oxygen and resource flux from the ambient fluid into the space confined by the streamers. To test this assumption, we first estimated the potential oxygen consumption time scale by bacteria within the streamer, *Τ*_us_, and compared it with the time scales for diffusive (*Τ*_d_) and advective (*Τ*_a_) delivery of oxygen into the gap to replenish oxygen consumption therein. Assuming a cellular respiration rate of 1 fmol O_2_ cell^−1^ h^−1^ [38, 39], *Τ*_u_ for 10^7^ cell ml^−1^ in the bulk fluid results in ~ 28 h. Be the uptake proportional to the concentration of active cells, a streamer, with an average porosity of 10% and thus a cell concentration in the order of 10^11^ cells ml^−1^, would locally decrease the uptake time scale by four orders of magnitude, yielding a *Τ*_us_ of ~ 10 s. Diffusion and advection depend on the distance traveled by oxygen, its diffusive coefficient, *D*, and on the fluid velocity of the bulk liquid. The diffusive time scale (*Τ*_d_ = *L*^2^/2*D*) over a length of half pore throat, *L*, equals 22 s. The advection time scale (*Τ*_a_ = *L*/*v*), given a mean velocity (*v*) in the gap of 0.01 mm s^−1^ equals 30 s. The time scales of oxygen diffusion and advection, which are ~ 2 and ~3 times higher, respectively, than *T*_us_, cannot satisfy the oxygen demand for bacteria within the space confined by the streamers (Fig. 5c). We argue that the contrasting time scales of oxygen replenishment and consumption induced the observed BB gap, which emerged from 70 h on (Fig. 5b), when streamers had reached significant coverage within the pore space (Fig. 4a). Furthermore, streamers were densely packed with metabolically active bacterial cells (Fig. S8 and S9).

To corroborate these theoretical considerations, we used 5-cyano-2,3-ditolyl tetrazolium chloride (CTC) to quantify the respiratory activity of bacterial cells in the streamers and in the BB remaining in the gap (see Biofilm respiratory activity in Supplementary material; Fig. S9). CTC is a redox-sensitive dye, which is reduced to the fluorescent and water insoluble formazan (CTF) within respiring bacterial cells [40]; CTC can be used therefore to detect and quantify respiration of single bacterial cells and clusters [41–44]. On average, we found significantly (*t* test; *p* < 0.001) lower respiratory activity associated with the little BB biomass remaining in gap than with the streamers (Fig. 5d). This is further evidence that the BB gap results from limited oxygen (and resource) availability.

### Architectural differentiation increases space exploitation

Does the gain of biomass in streamers outweigh the reduced biomass of BB in the gap? To address this question, we quantified the missing BB biomass in the gap for each time point (see Image processing in Supplementary material, Fig. S12) and added this to the observed BB biomass (Fig. 5e). This “gap filling” would add on average 16.5 ± 3.5% to BB. In contrast, the presence of streamers resulted in a net biomass gain of 25.5 ± 11.7%, and therefore architectural differentiation significantly increased the biomass gain (paired Wilcoxon signed-rank test, *p* < 0.01) (Fig. 5e). This insinuates that “gap filling” would be an inferior strategy to architectural differentiation to increase the carrying capacity of the biofilms in porous environments.

## Discussion

In analogy to the phenotypic plasticity of individual bacterial cells as a survival strategy [45], we evoke architectural plasticity [28] to contribute to the success of biofilms in porous environments. We found that hydrodynamics and related mass transfer mediated the differentiation of biofilms into an annular BB and streamers reproducibly across pore-grain complexes. Architectural differentiation was not paralleled by a differentiation of community composition, which highlights the architectural plasticity of the biofilm community. Thus, our findings suggest that architectural plasticity potentially enables multispecies biofilms to increase their carrying capacity in porous environments. This finding is noticeable given the limited pore space between grains and reduced mass transfer of nutrients and resources to the microorganisms—properties that typically make sedimentary environments more difficult to colonize than benthic or pelagic habitats.

Previous studies on biofilms in porous environments used monospecies systems [11, 12, 14, 21] as they rarely occur in natural (e.g., sediments, soils) or engineered systems (e.g., membranes). To mimic the diversity typically encountered in stream biofilms [4], we used a natural inoculum containing hundreds of OTUs. Architectural differentiation in the absence of marked differences in community composition contrasts previous findings on benthic biofilms [46]. This difference may be attributable to light and turbulent flow that instigate physical and chemical gradients, and hence niches for various bacterial taxa within benthic biofilms. The absence of such environmental cues may lead to the homogenization of bacterial taxa across the annular BB and streamers in multispecies biofilms forming in porous environments. This is the first evidence showing that architectural plasticity [28] occurs even within multispecies biofilms with hundreds of bacterial taxa. Prior to the inoculation of the fluidic devices, we have removed major grazers from the inoculum, potentially abundant in stream biofilms [47]; we recognize that this could be a simplification of an otherwise even more complex system. Nevertheless, our system with multispecies biofilms in heterogeneous flow landscapes certainly better reflects the physical conditions typically found in streambeds than the fluidic platforms traditionally used for monospecies biofilms [48].

We suggest that architectural plasticity allows biofilms to alter (or construct) their niche to the benefit of space exploitation by the entire community within the porous environment. In fact, the annular BB formed the basis for the growth of streamers that exploited the pore space beyond the range of BB and even beyond the range of their native local grain–pore complex. This underscores the complementary use of space as a resource in porous environments. Although streamers allow biofilms to expand space exploitation, they may be more susceptible to physical disturbance than BB. On the other hand, elevated resistance to flow of BB confers stability to the biofilm because it allows streamers to form de novo from the BB after sloughing. The high community similarity between streamers and BB facilitates this renewal. Architectural plasticity may thus be an example of an emergent property [24] promoting biofilm stability in porous environments.

Our findings suggest that different mechanisms underlie the exploitation of the space provided by the grain–pore complex by both biofilm architectures. The increase of the carrying capacity of BB was facilitated by tortuosity, which enlarges the biofilm interface to mass transfer of nutrients and resources. Similar relationships between biofilm topography (as tortuosity or fractality) and growth were previously reported from benthic biofilms in streams exposed to open-channel flow [3] and from mathematical models [49]. This appears particularly advantageous in the stagnant zones of porous environments, where diffusion governs mass transfer and often limits the delivery of nutrients, carbon, and oxygen.

Streamers reproducibly developed from the annular BB where fluid velocity was elevated in the throat between neighboring grains. Our model suggests that this initial phase of streamer growth was governed to a large extent by filtration of bacterial cells from the bulk liquid. The evidence from our model is further supported by the higher similarity between the bacterial community contained in the inoculum and the streamers, as compared to the similarity between the inoculum and BB. The filtration effect declined as streamer porosity and hence its permeability to fluid decreased. In fact, as porosity decreased, streamers increasingly resisted to the fluid flow thereby deviating its flow path (Fig. S4B) and transported bacterial cells. A similar interplay between streamer porosity and filtration of transported cells was reported from *P. aeruginosa* biofilms growing in meandering channels [17].

Our results suggest that streamer growth in length was constrained by the availability of physical space between the grains. Initially, streamer length exceeded the pore length scale (1.46 ± 0.18 mm) provided by the maximum distance within the pore space (that is, its diagonal). This was probably encouraged by open throats allowing streamers to extend downstream beyond the range of their native grain–pore complex. Reduction of the throat width owing to BB growth increased the probability of streamers to locally attach to BB, with consequences for their mechanical response (e.g., oscillation) to fluid flow mostly resulting in catastrophic disruption and downstream loss of the streamers. Their safe “range” within the local pore space was defined by the throat width (0.61 ± 0.13 mm) as the shortest length scale, which is indeed very close to the streamer length scale (0.59 ± 0.46 mm) at equilibrium. Taken together, this suggests that streamers in the laminar environment of porous systems contribute to the sustained exploitation of space as a resource, which is certainly facilitated by the viscoelastic property of their EPS backbone. Streamers developing in turbulent flow (such as in open channels) are thought as an adaptive response to high shear stress [20] and to increased mass transfer [50].

We found that constant flow better mimics the interface between water flow and benthic sediments in streams. Furthermore, we used relatively low substrate concentrations (3.4 ± 0.1 mg C L^−1^) as typically encountered in streams. This contrasts the optimal culture environment (e.g., tryptone broth) applied for monospecies biofilms forming copious streamers in porous devices [14, 15, 17]. Because of the constant flow and the low substrate concentrations used in this study, we did not reproduce streamer-induced clogging of pores as showed by previous studies [14, 17]. Therefore, our study yields insights in biofilm streamer dynamics in an environment that is similar to the environment found in the streambed at its interface to the open-channel flow.

Importantly, our results highlight that the differentiation of biofilms into a base component and streamers at the scale of individual grain–pore complexes benefits carrying capacity at the larger scale of the porous system, and hence biofilm fitness. However, this comes with a tradeoff at the cost of local BB growth downstream of the grains. Combining simple assumptions on oxygen mass transfer and uptake with imaging of biofilm respiratory activity, we present evidence that streamers inhibit local BB growth by locally reducing oxygen delivery to the bacterial cells in the stagnant zone. This notion complements previous results in turbulent regime, where streamers have been shown to sequester nutrients and resources from the fluid at high efficiency [50]. Our findings on bacterial respiratory activity (from CTC) suggest that this is also true for streamers in laminar flow.

To conclude, our study sheds new light on the interactions between biofilms and their physical environment in a porous system and hence on microbial ecology. Our findings suggest that architectural plasticity enables biofilms to differentiate into BB and streamers to complementarily exploit the space provided by the grain–pore complexes. Ultimately this increases the carrying capacity of biofilms, which is a cornerstone of the ecological success of any organism. This comes at a tradeoff between architectures, expanding the current view of tradeoffs (e.g., competition and cooperation) [51, 52] as drivers of spatially structured microbial communities.

## Accession number for sequence information

The number PRJEB31083 can be used to access our gene data and metadata on the European Nucleotide Archive.

## Supplementary information

Supplementary Material

## References

[1] Flemming HC, Wuertz S (2019). Bacteria and archaea on Earth and their abundance in biofilms. Nat Rev Microbiol.

[2] Prosser JI, Bohannan BJM, Curtis TP, Ellis RJ, Firestone MK, Freckleton RP (2007). The role of ecological theory in microbial ecology. Nat Rev Microbiol.

[3] Battin TJ, Kaplan LA, Newbold JD, Hansen CM (2003). Contributions of microbial biofilms to ecosystem processes in stream mesocosms. Nature.

[4] Battin TJ, Besemer K, Bengtsson MM, Romani AM, Packmann AI (2016). The ecology and biogeochemistry of stream biofilms. Nat Rev Microbiol.

[5] Lei G, Dong PC, Wu ZS, Gai SH, Mo SY, Li Z (2014). Multi-scale structures of porous media and the flow prediction. J Nat Gas Sci Eng.

[6] Gelhar L, Welty C, Rehfeldt K (2010). A critical review of data on field‐scale dispersion in aquifers. Water Resour Res.

[7] Cunningham AB, Characklis WG, Abedeen F, Crawford D (1991). Influence of biofilm accumulation on porous media hydrodynamics. Environ Sci Technol.

[8] Vandevivere P, Baveye P (1992). Saturated hydraulic conductivity reduction caused by aerobic bacteria in sand columns. Soil Sci Soc Am J.

[9] Thullner M, Mauclaire L, Schroth MH, Kinzelbach W, Zeyer J (2002). Interaction between water flow and spatial distribution of microbial growth in a two-dimensional flow field in saturated porous media. J Contam Hydrol.

[10] Stoodley P, Dodds I, De Beer D, Scott HL, Boyle JD (2005). Flowing biofilms as a transport mechanism for biomass through porous media under laminar and turbulent conditions in a laboratory reactor system. Biofouling.

[11] Valiei A, Kumar A, Mukherjee PP, Liu Y, Thundat T (2012). A web of streamers: biofilm formation in a porous microfluidic device. Lab Chip.

[12] Nadell CD, Ricaurte D, Yan J, Drescher K, Bassler BL (2017). Flow environment and matrix structure interact to determine spatial competition in Pseudomonas aeruginosa biofilms. eLife.

[13] Borer B, Tecon R, Or D (2018). Spatial organization of bacterial populations in response to oxygen and carbon counter-gradients in pore networks. Nat Commun.

[14] Hassanpourfard M, Ghosh R, Thundat T, Kumar A (2016). Dynamics of bacterial streamers induced clogging in microfluidic devices. Lab Chip.

[15] Rusconi R, Lecuyer S, Guglielmini L, Stone HA (2010). Laminar flow around corners triggers the formation of biofilm streamers. J R Soc Interface.

[16] Rusconi R, Lecuyer S, Autrusson N, Guglielmini L, Stone HA (2011). Secondary flow as a mechanism for the formation of biofilm streamers. Biophys J.

[17] Drescher K, Shen Y, Bassler BL, Stone HA (2013). Biofilm streamers cause catastrophic disruption of flow with consequences for environmental and medical systems. Proc Natl Acad Sci.

[18] Coyte KZ, Tabuteau H, Gaffney EA, Foster KR, Durham WM (2017). Microbial competition in porous environments can select against rapid biofilm growth. Proc Natl Acad Sci.

[19] Stoodley P, Dodds I, Boyle JD, Lappin-Scott H (1998). Influence of hydrodynamics and nutrients on biofilm structure. J Appl Microbiol.

[20] Stoodley P, Cargo R, Rupp CJ, Wilson S, Klapper I (2002). Biofilm material properties as related to shear-induced deformation and detachment phenomena. J Ind Microbiol Biotechnol.

[21] Marty A, Roques C, Causserand C, Bacchin P (2012). Formation of bacterial streamers during filtration in microfluidic systems. Biofouling.

[22] Niederdorfer R, Peter H, Battin TJ (2016). Attached biofilms and suspended aggregates are distinct microbial lifestyles emanating from differing hydraulics. Nat Microbiol.

[23] Scott‐Phillips Thomas C, Laland Kevin N, Shuker David M, Dickins Thomas E, West SA (2014). The niche construction perspective: a critical appraisal. Evolution.

[24] Flemming HC, Wingender J, Szewzyk U, Steinberg P, Rice SA, Kjelleberg S (2016). Biofilms: an emergent form of bacterial life. Nat Rev Microbiol.

[25] An D, Danhorn T, Fuqua C, Parsek MR (2006). Quorum sensing and motility mediate interactions between Pseudomonas aeruginosa and Agrobacterium tumefaciens in biofilm cocultures. Proc Natl Acad Sci.

[26] Rendueles O, Ghigo J-M. Mechanisms of competition in biofilm communities. Microbiol Spectr. 2015; 3:MB-0009-2014. 10.1128/microbiolspec.MB-0009-2014 26185066

[27] Liu W, Røder HL, Madsen JS, Bjarnsholt T, Sørensen SJ, Burmølle M (2016). Interspecific bacterial interactions are reflected in multispecies biofilm spatial organization. Front Microbiol.

[28] Bridier A, Piard JC, Pandin C, Labarthe S, Dubois-Brissonnet F, Briandet R (2017). Spatial organization plasticity as an adaptive driver of surface microbial communities. Front Microbiol.

[29] Jessup CM, Kassen R, Forde SE, Kerr B, Buckling A, Rainey PB (2004). Big questions, small worlds: microbial model systems in ecology. Trends Ecol Evol.

[30] Yawata Y, Nguyen J, Stocker R, Rusconi R (2016). Microfluidic studies of biofilm formation in dynamic environments. J Bacteriol.

[31] Aleklett K, Kiers ET, Ohlsson P, Shimizu TS, Caldas VE, Hammer EC (2018). Build your own soil: exploring microfluidics to create microbial habitat structures. ISME J.

[32] Krause S, Lewandowski J, Grimm NB, Hannah DM, Pinay G, McDonald K (2017). Ecohydrological interfaces as hot spots of ecosystem processes. Water Resour Res.

[33] Kim K, Drescher K, Shun Pak O, Bassler BL, Stone HA (2014). Filaments in curved streamlines: rapid formation of Staphylococcus aureus biofilm streamers. New J Phys.

[34] Hassanpourfard M, Nikakhtari Z, Ghosh R, Das S, Thundat T, Liu Y (2015). Bacterial floc mediated rapid streamer formation in creeping flows. Sci Rep.

[35] Brumley DR, Polin M, Pedley TJ, Goldstein RE (2015). Metachronal waves in the flagellar beating of Volvox and their hydrodynamic origin. J R Soc Interface.

[36] Caporaso JG, Lauber CL, Walters WA, Berg-Lyons D, Lozupone CA, Turnbaugh PJ (2011). Global patterns of 16S rRNA diversity at a depth of millions of sequences per sample. Proc Natl Acad Sci.

[37] Edgar Robert C. (2010). Search and clustering orders of magnitude faster than BLAST. Bioinformatics.

[38] Gong X, Garcia-Robledo E, Schramm A, Revsbech NP (2016). Respiratory kinetics of marine bacteria exposed to decreasing oxygen concentrations. Appl Environ Microbiol.

[39] Warkentin M, Freese HM, Karsten U, Schumann R (2007). New and fast method to quantify respiration rates of bacterial and plankton communities in freshwater ecosystems by using optical oxygen sensor spots. Appl Environ Microbiol.

[40] Rodriguez GG, Phipps D, Ishiguro K, Ridgway HF (1992). Use of a fluorescent redox probe for direct visualization of actively respiring bacteria. Appl Environ Microbiol.

[41] Gasol J, Arístegui J (2007). Cytometric evidence reconciling the toxicity and usefulness of CTC as a marker of bacterial activity. Aquat Microb Ecol.

[42] Smith E (1998). Coherence of microbial respiration rate and cell-specific bacterial activity in a coastal planktonic community. Aquat Microb Ecol.

[43] Sherr B, del Giorgio P, Sherr E (1999). Estimating abundance and single-cell characteristics of respiring bacteria via the redox dye CTC. Aquat Microb Ecol.

[44] Cook KL, Garland JL (1997). The relationship between electron transport activity as measured by CTC reduction and CO2 production in mixed microbial communities. Microb Ecol.

[45] Justice SS, Hunstad DA, Cegelski L, Hultgren SJ (2008). Morphological plasticity as a bacterial survival strategy. Nat Rev Microbiol.

[46] Besemer K, Hödl I, Singer G, Battin TJ (2009). Architectural differentiation reflects bacterial community structure in stream biofilms. ISME J.

[47] Weitere M, Erken M, Majdi N, Arndt H, Norf H, Reinshagen M (2018). The food web perspective on aquatic biofilms. Ecol Monogr.

[48] Bjarnsholt T, Alhede M, Alhede M, Eickhardt-Sørensen SR, Moser C, Kühl M (2013). The in vivo biofilm. Trends Microbiol.

[49] Picioreanu C, van Loosdrecht MCM, Heijnen JJ (1998). Mathematical modeling of biofilm structure with a hybrid differential-discrete cellular automaton approach. Biotechnol Bioeng.

[50] Taherzadeh D, Picioreanu C, Küttler U, Simone A, Wall WA, Horn H (2010). Computational study of the drag and oscillatory movement of biofilm streamers in fast flows. Biotechnol Bioeng.

[51] Nadell CD, Drescher K, Foster KR (2016). Spatial structure, cooperation and competition in biofilms. Nat Rev Microbiol.

[52] Ghoul M, Mitri S (2016). The ecology and evolution of microbial competition. Trends Microbiol.

