# Supplementary material for: Unraveling the biophysical underpinnings to the success of multispecies biofilms in porous environments
Source: ISME J. 2019 Mar 4;13(7):1700–10. doi: 10.1038/s41396-019-0381-4 (PMC6776110; doi:10.1038/s41396-019-0381-4)
Supplement: Supplementary file 1 — Supplementary Material [file 41396_2019_381_MOESM1_ESM.docx]

**Supplementary Material for**

**Unraveling the biophysical underpinnings to the success of multispecies biofilms in porous environments**

David Scheidweiler^1^, Hannes Peter^1^, Paraskevi Pramateftaki^1^, Pietro de Anna^2^, and Tom J. Battin^1*^

^1^ Stream Biofilm and Ecosystem Research Laboratory, Ecole Polytechnique Fédérale de Lausanne, CH-1015 Lausanne, Switzerland

^2^ Institute of Earth Sciences, University of Lausanne, CH-1015 Lausanne, Switzerland

*Corresponding author: [tom.battin@epfl.ch](mailto:tom.battin@epfl.ch)

**Supplementary Materials and Methods**

***Image processing***

To extract biofilm architectural information, we developed Matlab scripts to recursively analyze the collected large images. To determine the time series of biofilm areal coverage — as a proxy for biomass — we used the first image (T = 0 h) without biofilm as background. This background was subtracted from each subsequent image, accounting for shading correction. The difference was transformed into a binary matrix, where each pixel above 0 was set to 1; we interpreted this as biomass absorbing light. We defined BB as the fraction of biofilm present in a virtual ring around a grain whose internal boundary is the grain perimeter and the external boundary is defined such that the at least a fraction, γ, of the ring area is covered by biomass (Supplementary Fig. S10A and B*).* We tested three γ thresholds (25, 35 and 45 %), which resulted in slightly different coverage of BB than streamer biomass, however, without changing the observed patterns and conclusions (Supplementary Fig. S10C-E). For the results discussed here we have chosen the value γ = 35 %. We derived all architectural features (e.g., biofilm coverage, BB thickness and tortuosity, streamer width and length) from these matrices. Streamers were identified and quantified as the difference between the total biofilm and BB. Biofilm coverage was extracted from the binary images as the sum of the biofilm pixels of known physical size. For each grain, BB thickness was determined with 0.1° increments around each grain (Fig 4A). The BB perimeter, *P_BB_,* was computed as the sum of the Euclidean distance between neighboring points of its external boundary. Tortuosity was measured as the ratio between the measured *P_BB_* and the grain perimeter (Supplementary Fig. S10F) [1].

In bright-field microscopy, streamers initially occurred as individual small clusters likely connected by a non-visible EPS filament. To define the streamer boundary in the binary image, all clusters distant less than a tenth of pore throat (that is, 45 µm) were connected to each other by a one-pixel line into a single cluster (Supplementary Fig. S11A and B). This scale was large enough to capture the whole streamer but small enough to avoid mixing streamers arising from different locations. Next, each string of connected pixels was labeled as a streamer. In order to remove the small clusters of biofilm growing on the bottom of the fluidic chamber, all the objects with surface smaller than the fifth percentile were removed from data analyses. Streamer porosity was measured as the ratio between the total number of pixels (Supplementary Fig. S11B) and the number of pixels identifying biomass (*Fig. S11A*), within each streamer boundary. Streamers were treated by erosion image processing consisting in the generation of a skeleton with a one-pixel width [2]. Streamer length (*L*) was measured as the longest connected path within the streamer skeleton (Supplementary Fig. S11C), whereas streamer width (*W*) was derived from Euclidean distance transform [2]. This process measures the local distance from the external boundary to the defined internal longest connected path (Supplementary Fig. S11D), here termed streamer radius (*R_S_*). The streamer *W* was computed as the average over all the *n* pixels *k* composing the defined streamer length:

|  | $W=(\frac{\sum_{k=1}^{n} {(2 R}_{S,k}).}{n}).$ | (1) |
| --- | --- | --- |

In order to estimate the gap in the BB, we measured the surface of a circular sector (*S*) characterized by a subtended angle of 100°, chosen based on the gap boundaries (Fig. 5B) and radius equal to the sum of the grain radius and the maximum thickness of BB within the region of the gap. We then subtracted the circular sector area occupied by the grain (*S_G_*) and by the biofilm layer in the gap region (*S_BB_*) from this area (Supplementary Fig. S12). The gap filling is the result of the sum of all grain gaps.

***Carrying capacity***

The carrying capacity of biofilm at the level of the entire fluidic device and for individual BB on the grains (in number of 200) was determined fitting a logistic growth model with nonlinear regressions [4] to the BB measured biomass at time t, *BB(t)*.

|  | $BB\left( t \right)=\left( \frac{K}{1+e^{-r\left( t-t_{0} \right)}} \right),$ | (2) |
| --- | --- | --- |

where *K* is the carrying capacity and *r* the maximum growth rate. The normalized carrying capacity, *nK*, is the ratio between the carrying capacity, *K*, and the biomass once the grain was fully covered by biofilm.

***Filtration model***

We expect each individual streamer to increase in width *W* in function of the amount of transported particles filtered by the streamer. For simplicity we consider the streamer as a permeable cylinder orthogonally oriented to the flow direction and whose cross-sectional area, *A_s_*, changes as:

|  | $\frac{dAs}{dt}=\frac{d\pi W^{2}}{4dt}=2\pi W\frac{dW}{4dt}=\beta v C W V$ | (3) |
| --- | --- | --- |
|  | $W=\frac{2\beta v C V t}{\pi}$ | (4) |

where *β* is the fraction of cells intercepted by the streamer (see below), *v* is fluid average velocity, *C* is cell concentration in the bulk liquid, *V* is the average transported cell volume and *t* is time. Confidence intervals of the model were determined by standard error propagation of uncertainty[5].

The fraction of cells caught in the streamer, *β*, was measured in an independent experiment. During initial biofilm growth over 5 days, we injected every 24 h an amount of fluorescent particles (*P_i_*) (1 µm; Thermofisher Fluoromax B0100) into fluidic device growth, calculated as

|  | $P_{i}=C_{p}Q T,$ | (5) |
| --- | --- | --- |

where *C_p_* is particle concentration, *Q* is flow rate and *T* is the duration of the injection. We then determined the actual amount of particles passing through the individual streamers (*P_is_*) by multiplying *P_i_* by the ratio of streamer surface (*A_s_*) and pore transverse cross section (*A_p_*)

|  | $P_{is}=P_{i} (\frac{As}{Ap})$ | (6) |
| --- | --- | --- |

We evaluated the amount of fluorescent particles retained in the streamers (*P_s_*) from the difference between two images taken before and after the fluorescent particles injection, and then determined *β* as the ratio between *P_s_* and *P_is_*.

***Biofilm respiratory activity***

Respiratory activity was assessed using 5-cyano-2,3-ditolyl tetrazolium chloride (CTC), which is reduced into the fluorescent formazan (CTF) by respiring cells [7]. At the end of the experiment, a solution of CTC was injected directly in the fluidic device ensuring a 30-min residence time and a final 5 mM concentration. After rinsing the fluidic device with sterile (0.22 µm) streamwater for 60 min, biofilms were imaged with epifluorescence microscopy (Zeiss filter set 63 HE; excitation 572/25 nm, emission 629/62 nm, beam splitter 590 nm) with an exposure time of 100 ms per image. The relative fluorescence intensity of CTF was normalized by unit surface for both streamers and BB within the gap.

***Pseudomonas putida KT2440 culture***

Cultures of *P. putida* KT2440 expressing the green fluorescent protein (GFP) were grown overnight in 5 mL LB media at 30°C while shaking at 250 rpm. The culture was than flushed at 0.2 mL min^-1^ through the fluidic device at a concentration of ~10^7^ cell mL^-1^. *P. putida* KT2440 was a gift from A. Dechesne (Technical University of Denmark).

**Supplementary figures**


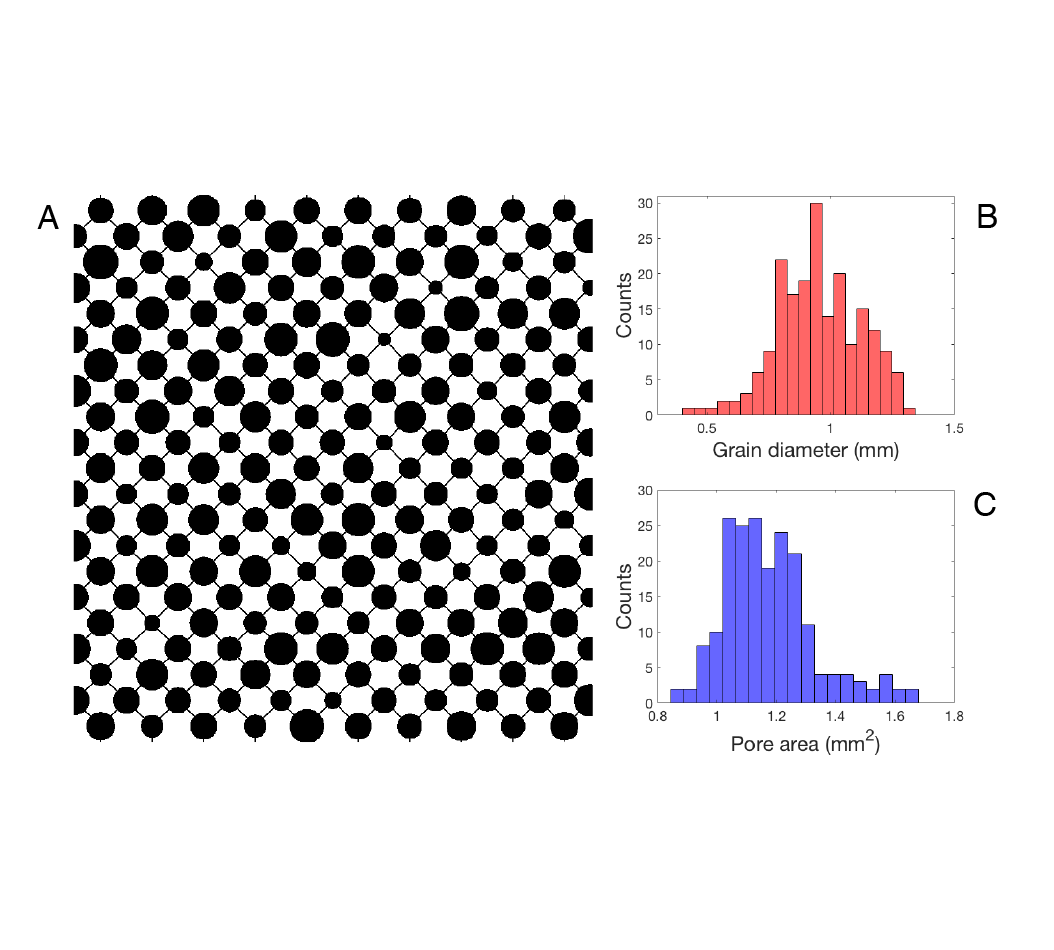


**Fig. S1.** **Porous system geometry.** (**A**) A synthetic 2d porous system was produced with 200 grains dispersed on a regular lattice matrix and characterized by a porosity ϕ = 0.60. (***B-C***) To promote flow heterogeneity the porous system was designed by varying grain radii resulting in normally distributed pore sizes.


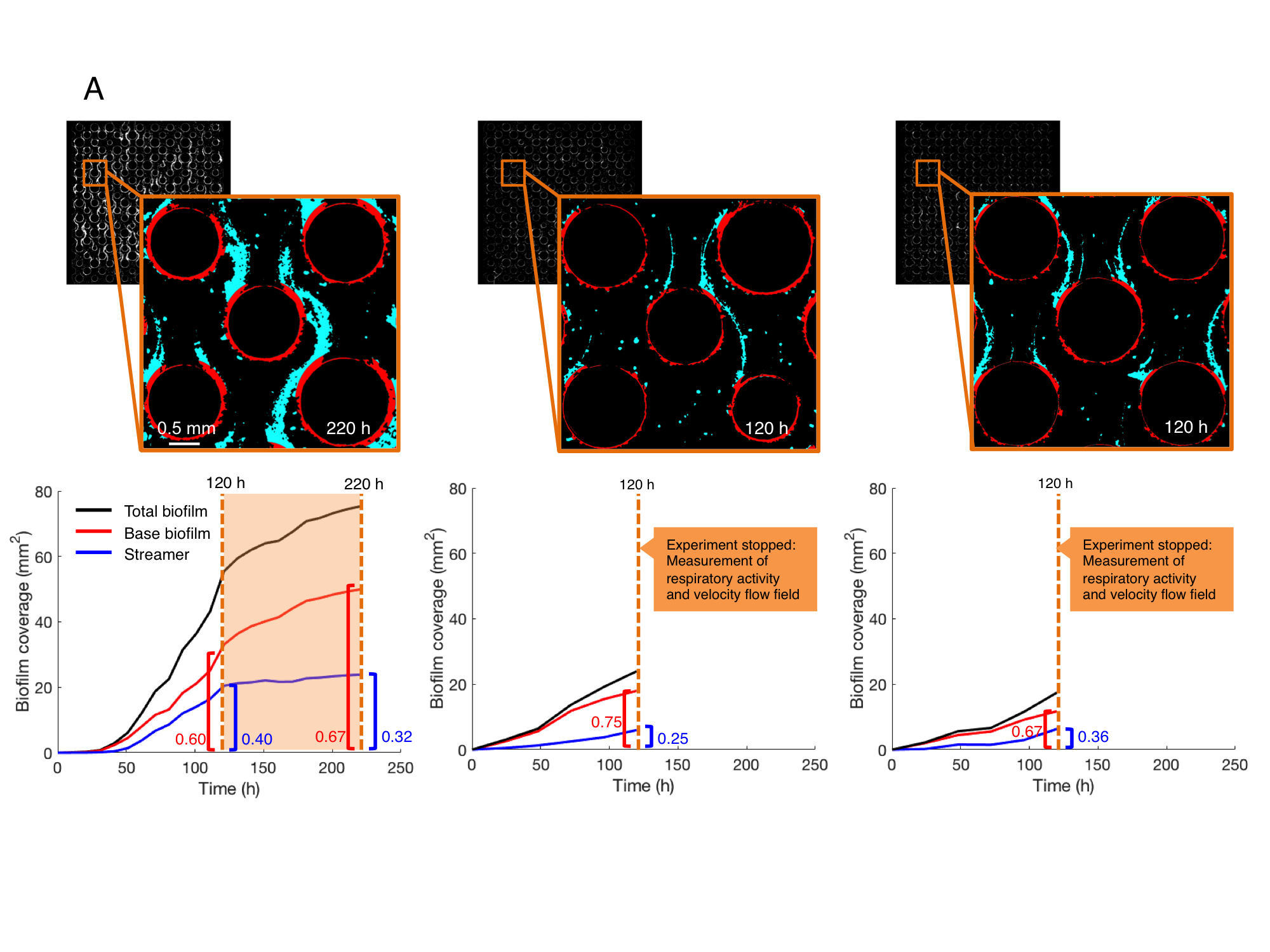

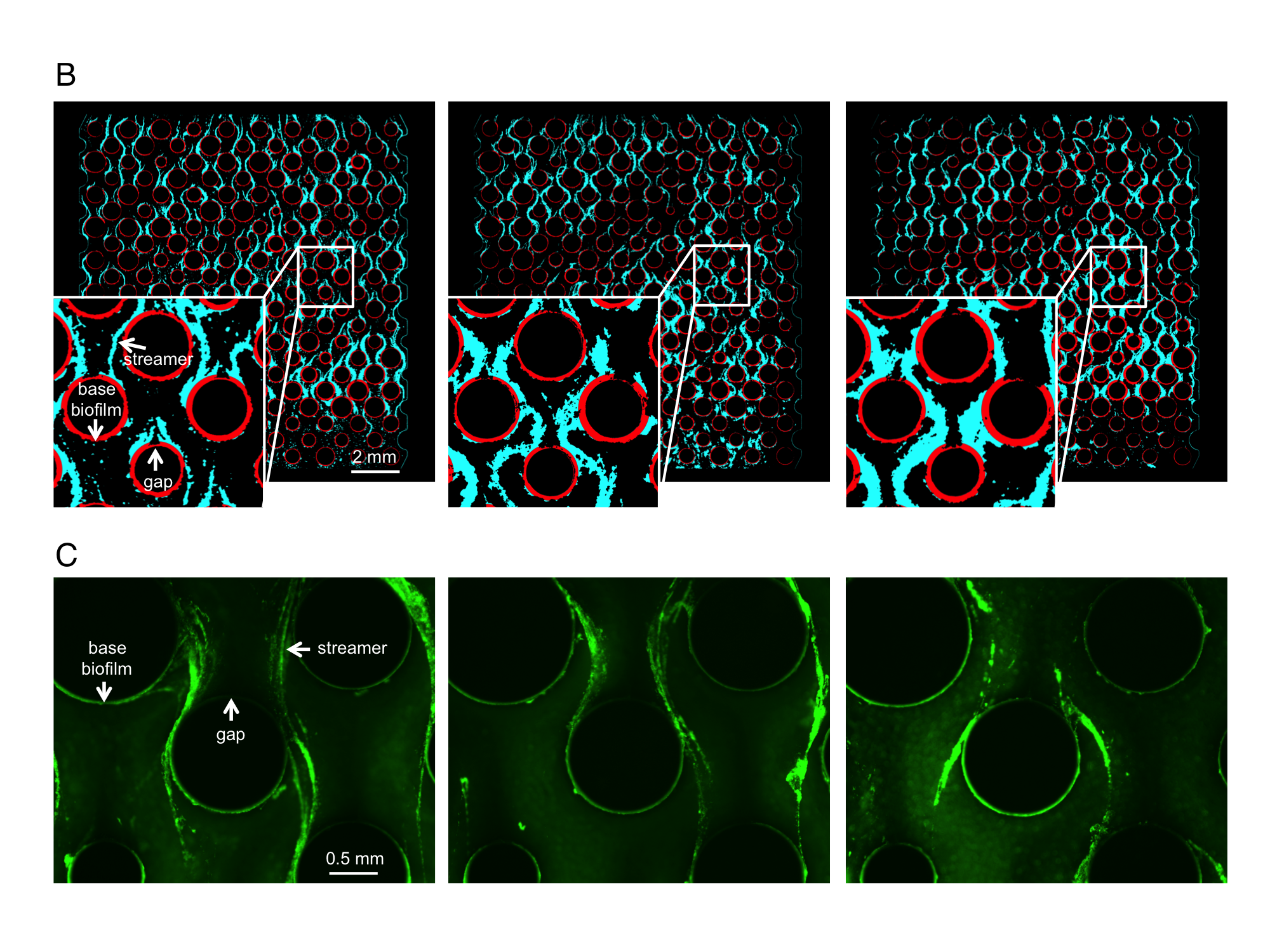


**Fig. S2.** **Replicability and system specificity.** (**A**) A first experiment conducted with stream water showed a differentiation in base biofilm (BB, in red) and streamer architectures (cyan). The total fractions of BB and streamer is reported in red and blue respectively, and in all replicates BB contributed more to the total biofilm biomass (BB = 67±6% Streamer = 34±6%) at 120h. The experiment has been repeated with different inocula: (**B**) lake water (Lake Geneva, Lausanne) processed exactly as the streamwater experiment, and (**C**) the model bacterium *P. putida* KT2440 which expresses green fluorescent protein. See Supplementary material, *Pseudomonas putida KT2440 culture*.


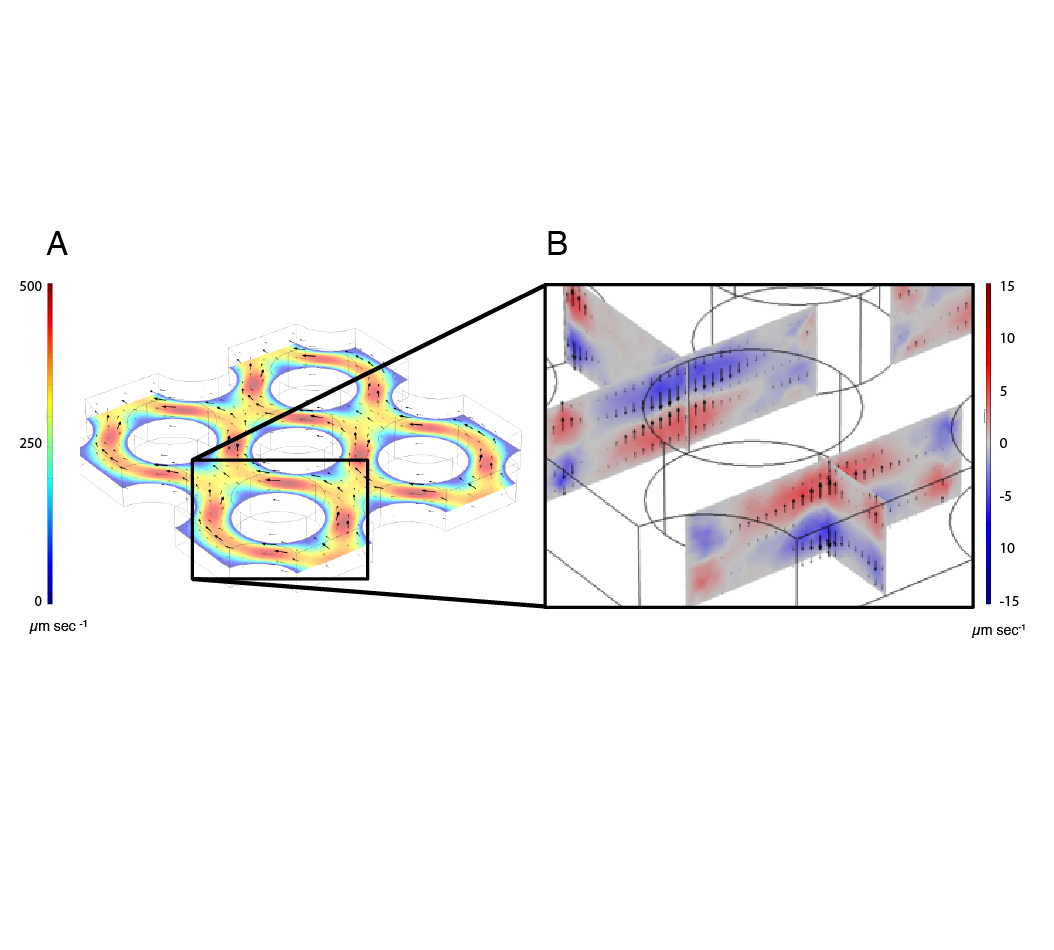


**Fig. S3.** **Flow simulations.** Flow was numerically solved using COMSOL Multiphysics on a finite element formulation and applying the three-dimensional Navier-Stokes equations, using portion of the experimental device’s 3D porous geometry and the imposed flow rate. (**A**) Shown is the velocity field of primary flows in a section at half height of the porous system. (**B**) The velocity fields in z direction at grain poles are shown. Velocity vectors in both panels are represented by arrows aligned as the local flow direction and length proportional to the velocity amplitude that is also represented by the colormap.

**
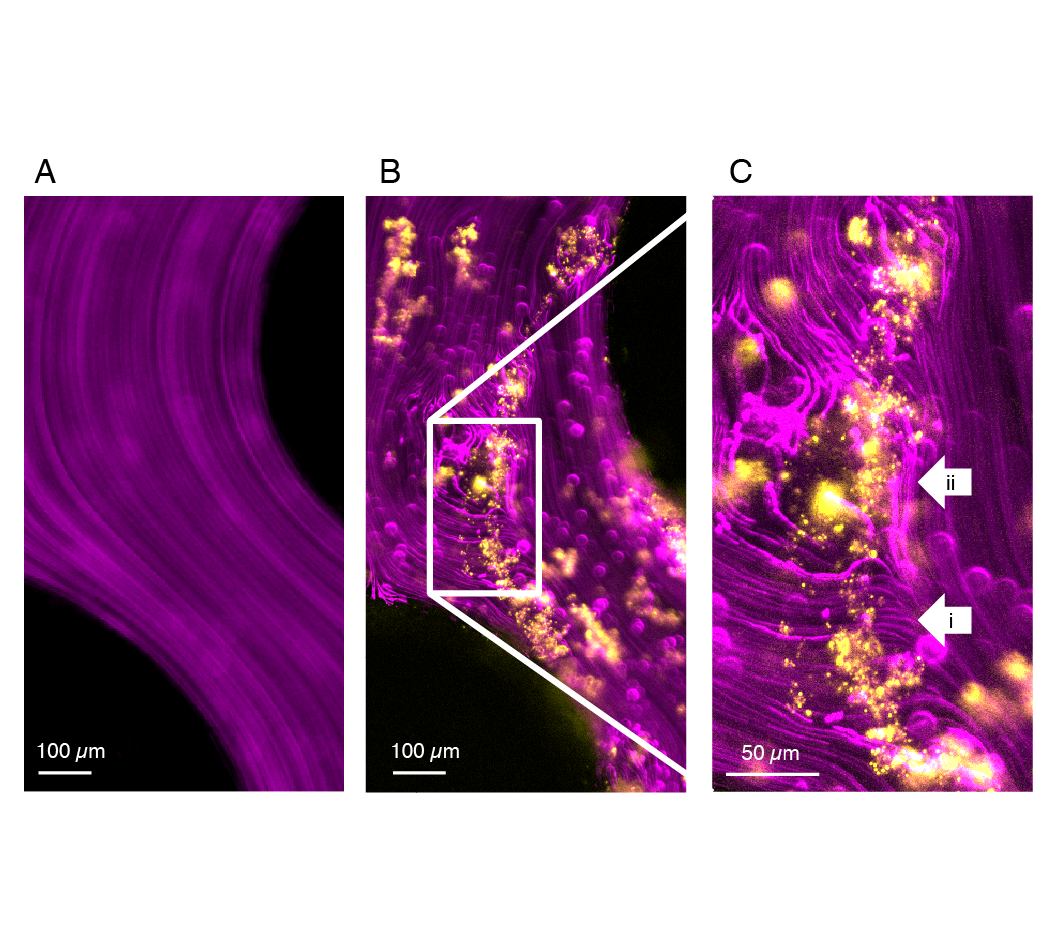
**

**Fig. S4.** **Streamer during exposure to neutrally buoyant fluorescent beads.** (**A**) Trajectory of transported fluorescent beads, shown in purple, prior to biofilm establishment. (**B**) Trajectory of transported fluorescent beads, shown in purple, on top of active respiring cells stained with CTC, in yellow. (**C**) Close up, showing area where the streamer is permeable to the flow (i), and a denser spot where the flow get deviated by the biomass (ii).


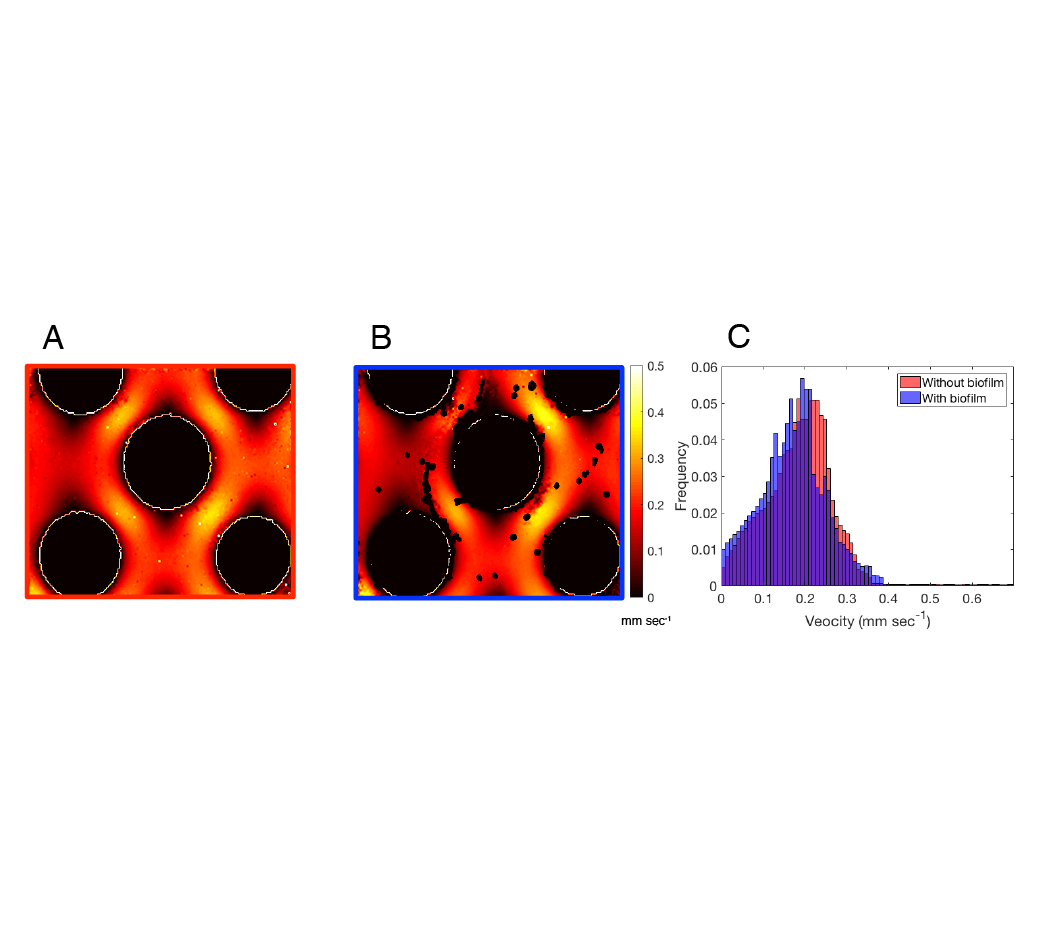


**Fig. S5.** **Biofilm impact on hydrodynamics.** The flow field was characterized in absence (**A**) and presence (**B**) of biofilms in the porous system. It results evident from the distribution of velocities (**C**) that the flow field was only marginally affected by the biofilm growth.

**Fig. S6.** **Bacterial community composition was similar in streamer and base biofilm.** (**A**) This illustration depicts OTUs as circles with lines connecting them to the respective samples (diamonds) in which they were detected. The relative abundance in a respective sample type is indicated by the width of the grey lines. Most OTUs (color shows taxonomic affiliation and circle size indicates overall relative abundance) occurred in both biofilm architectures and the inoculum (in the center of the graph). (**B**) Both biofilm communities were similarly recruited from the inoculum with 96.1% of the abundant OTUs (> 10 reads) in BB and 98.3% of abundant OTUs in streamers being found in the inoculum. Most of these OTUs were detected in similar relative abundance in the biofilm and inoculum communities, however, those that were enriched in biofilms compared to the inoculum were enriched to similar degrees in BB and streamers.


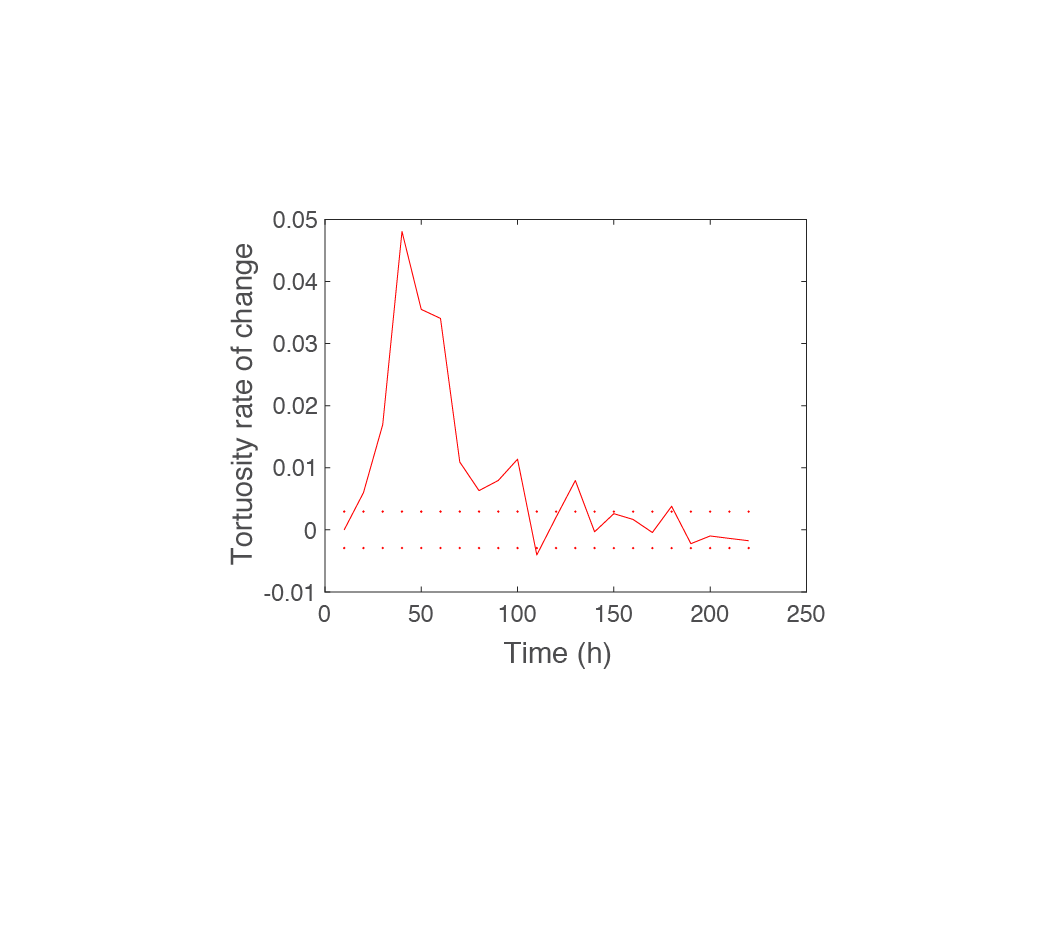


**Fig. S7**. **Tortuosity rate of change.** The rate of change of tortuosity T peaked till 50 h once a thin layer of base biofilm covered the grains and then approached a steady state (i.e. dT/dt ~ 0). The red dashed lines delimitate the standard error around zero.


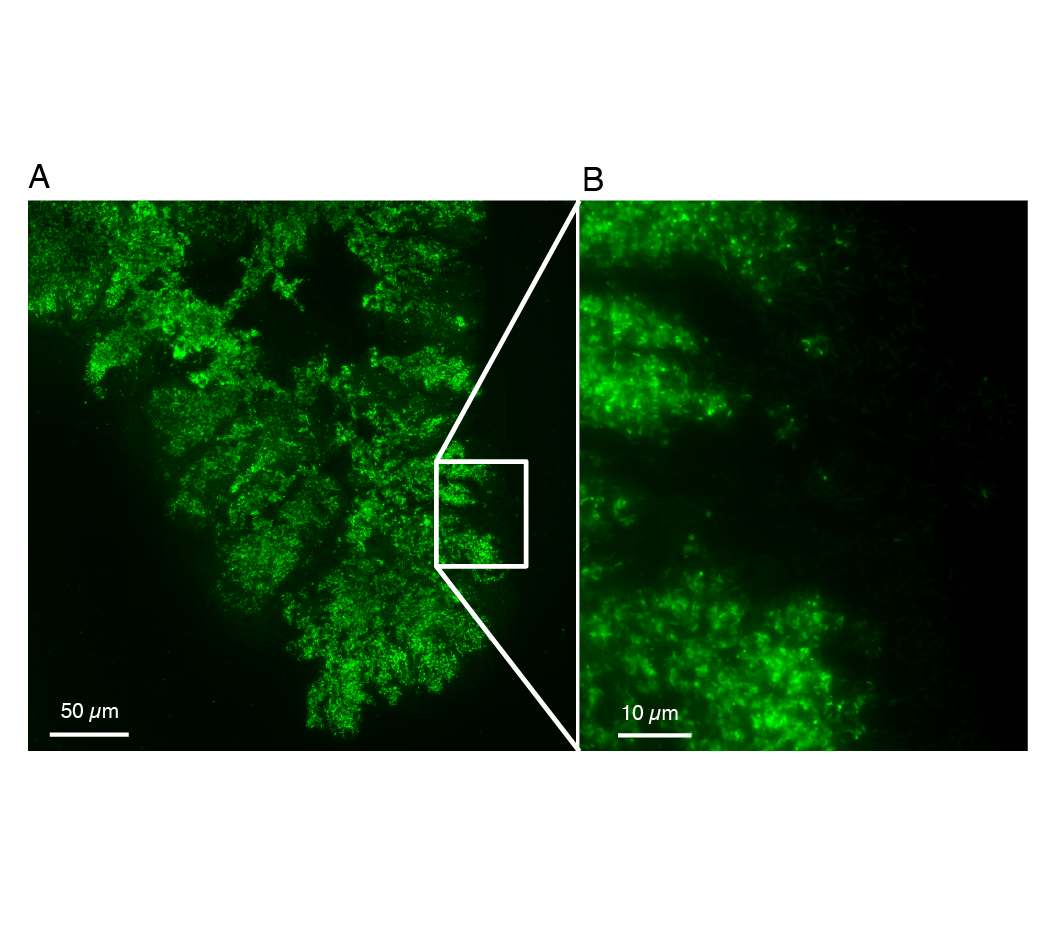


**Fig. S8**. **Bacterial cells within streamers.** Epifluorescence microscopy (40X magnification) shows densely packed bacterial cells in the tip of a streamer (220 h), potentially also containing extracellular DNA as this is also stained by SYTO13 dye (Life Technologies).


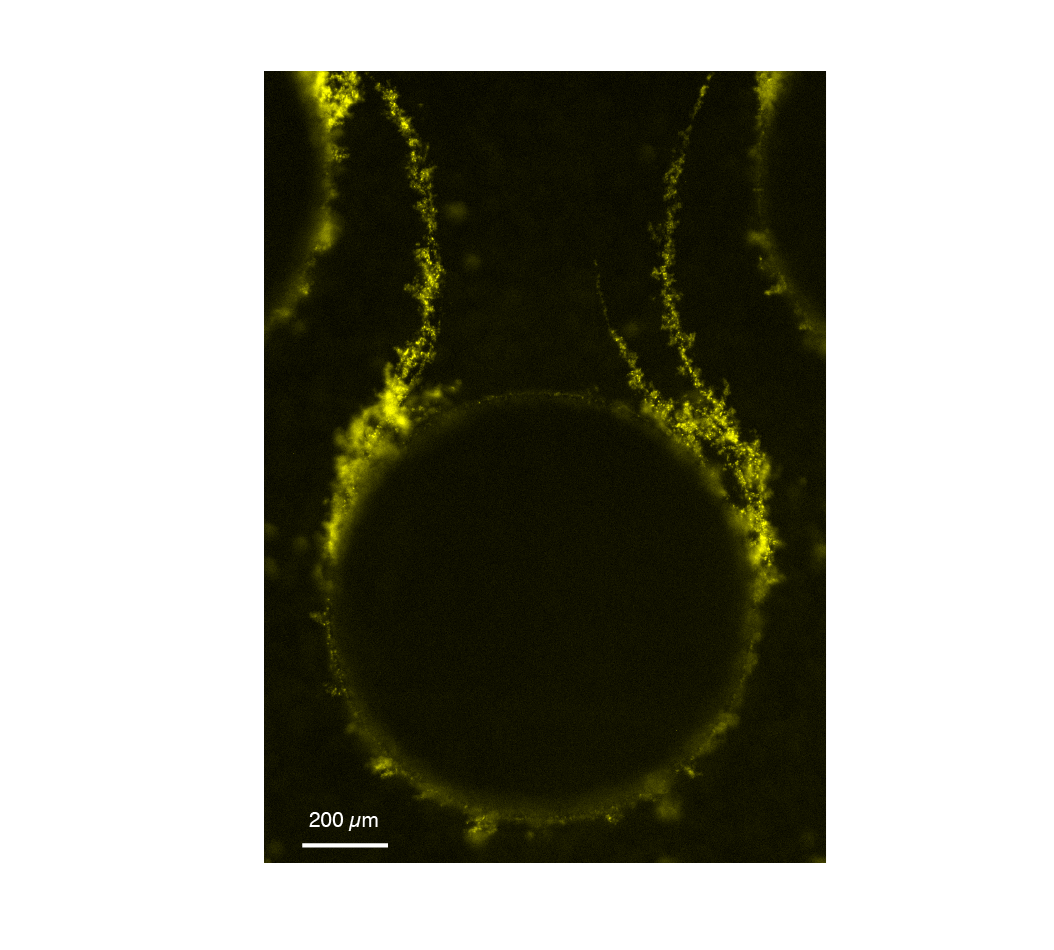


**Fig. S9.** **Bacterial respiratory activity.** 5-cyano-2,3-ditolyl tetrazolium chloride (CTC) staining was used to quantify respiratory activity of streamer and base biofilms after 120 h. The intensity of the fluorescent reaction, which is the result of formazan production by the reduction of CTC (in yellow), is directly proportional to the bacterial cell respiration.


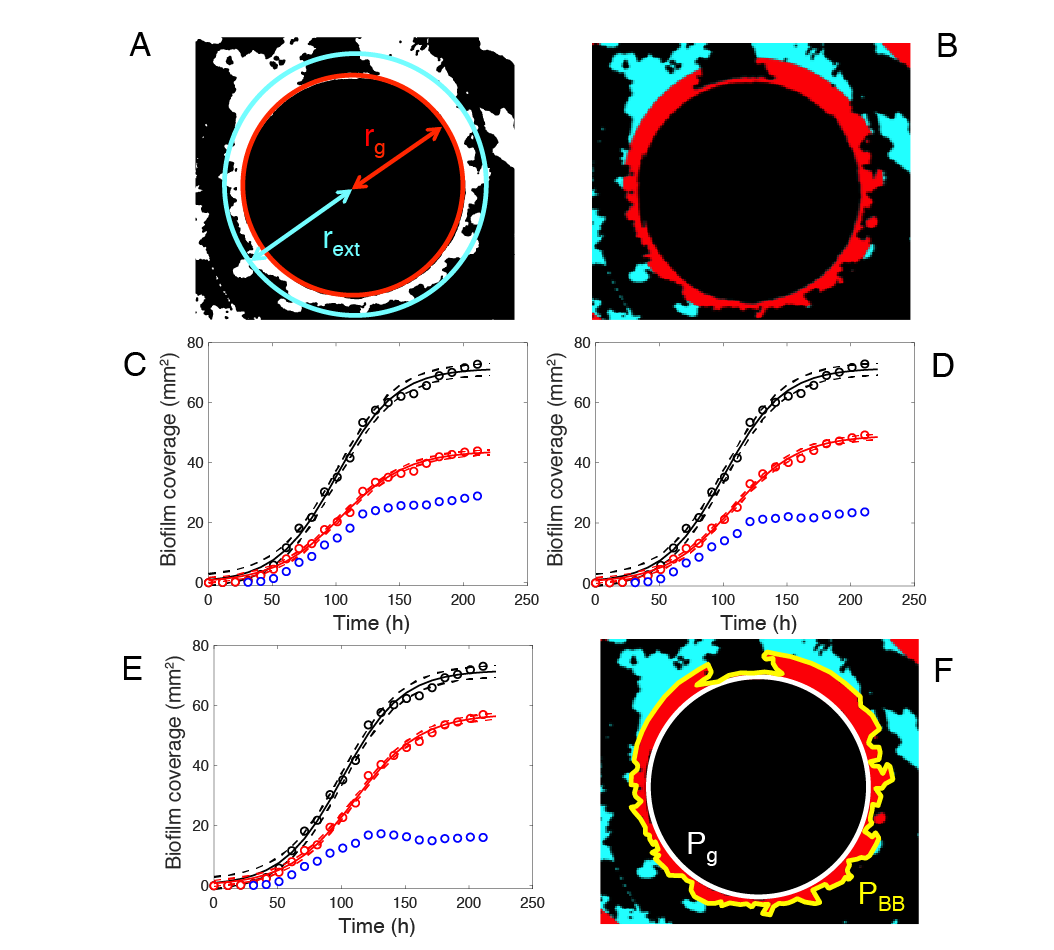


**Fig. S10. Discretization of biofilm architectures.** (**A-B**) We defined the base biofilm (BB) as the fraction of biofilm present in a virtual ring around a grain, whose internal boundary is the grain perimeter (red circle) and the external boundary (blue circle) is defined such that BB / (π (r_g_^2^ - r_ext_^2^ )) ≥ γ. Where γ is the threshold, while r_g_ and r_ext_ are respectively the grain and external boundary radii. Different thresholds, γ, were explored: 45% (**C**), 35% (**D**), and 25% (E), however the overall dynamics and conclusions did not change. They all consistently showed higher coverage of BB than streamer biomass. (**F**) Tortuosity was determined as the ratio between the measured BB perimeter (P_BB_) and the grain perimeter (P_g_).

**
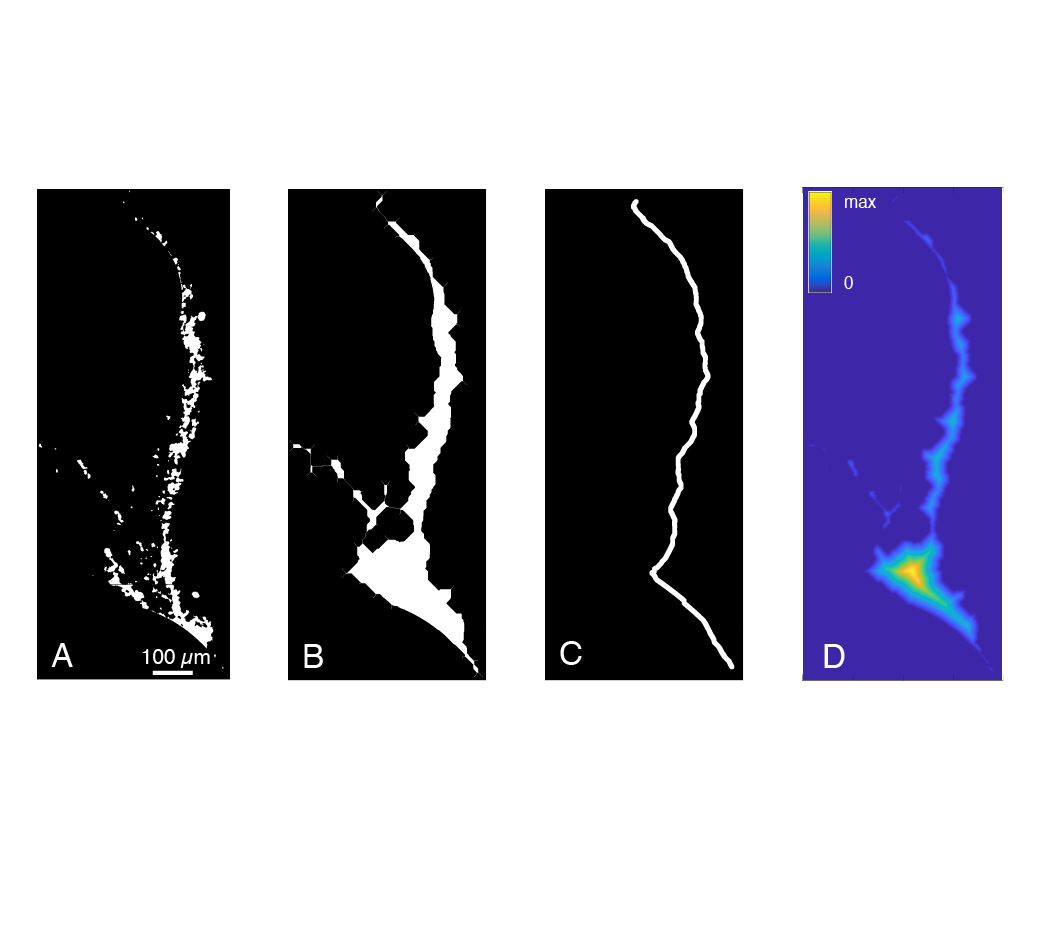
**

**Fig. S11**. **Determination of streamer architectural parameters.** (A) Streamers appeared as unconnected fragments aligned along flow streamlines. (B) Streamer fragments within 45 µm distance were connected by sequentially dilating and eroding the images. (C) The individual streamer length was estimated using the skeletonized image and measuring the longest connected path within a single skeleton (39). (D) Euclidean distance transform shows the distance from the external boundary toward the internal longest connected path (represented by the colormap). The average of the pixel values, which lay on the longest connected path, represents the streamer radius dimension.

**
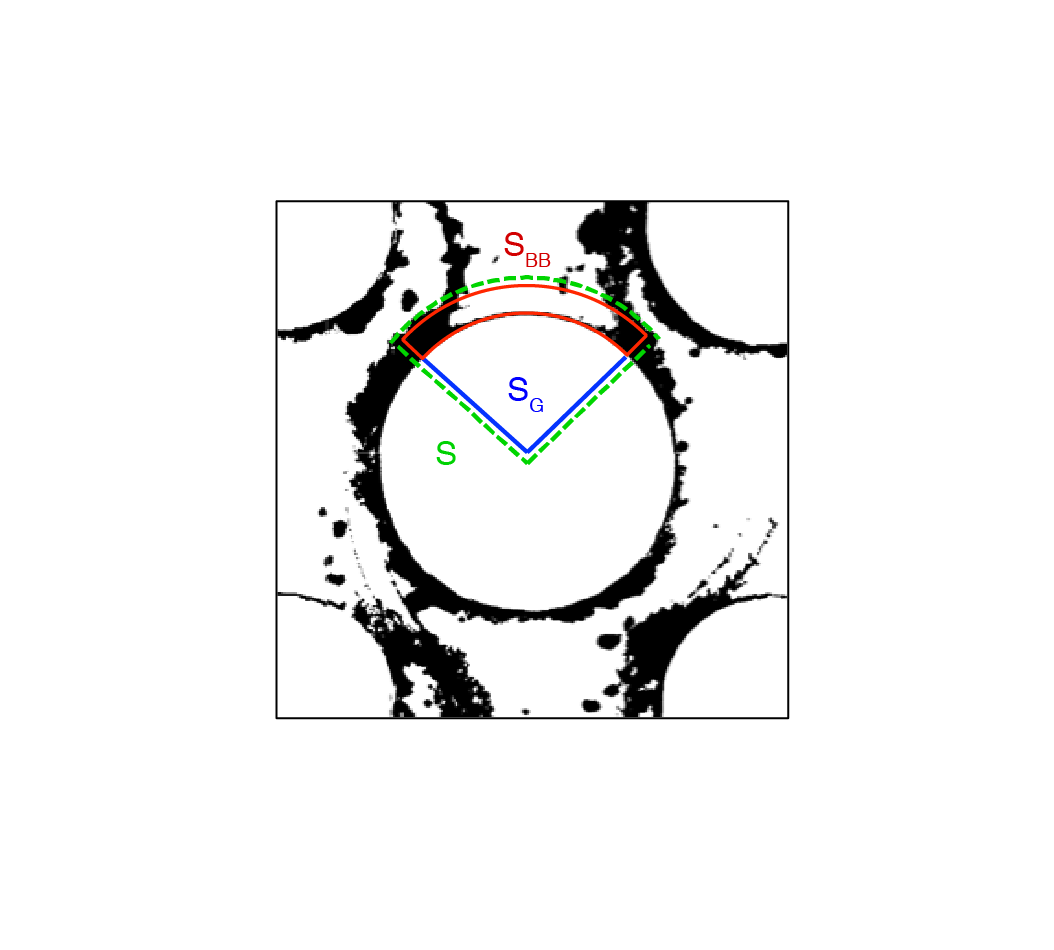
**

**Fig. S12.** Gap estimation. To quantify the areal extent of the gap in the BB, we measured the surface of a circular sector (*SBB*) between 130° and 230° (the pole being at 180°) and the maximum thickness of BB within the region of the gap. The angular extent of the gap was obtained from Fig. 4B in the main text.

**Supplementary references**

1. Battin TJ, Kaplan LA, Newbold JD, Hansen CM. Contributions of microbial biofilms to ecosystem processes in stream mesocosms. *Nature* 2003; **426**: 439–442.

2. Gonzalez RC. Digital Image Processing. 2009. Pearson Education.

3. Brumley DR, Polin M, Pedley TJ, Goldstein RE. Metachronal waves in the flagellar beating of Volvox and their hydrodynamic origin. *J R Soc Interface* 2015; **12**: 20141358.

4. Cavallini F. Fitting a Logistic Curve to Data. *Coll Math J* 1993; **24**: 247.

5. Taylor JR. An Introduction to Error Analysis: The Study of Uncertainties in Physical Measurements. 1982. Univ. Press, Oxford.

6. Caporaso JG, Lauber CL, Walters WA, Berg-Lyons D, Lozupone CA, Turnbaugh PJ, et al. Global patterns of 16S rRNA diversity at a depth of millions of sequences per sample. *Proc Natl Acad Sci* 2011; **108**: 4516–4522.

7. Rodriguez GG, Phipps D, Ishiguro K, Ridgway HF. Use of a fluorescent redox probe for direct visualization of actively respiring bacteria. *Appl Environ Microbiol* 1992; **58**: 1801–1808.
